# Self-Reported Effectiveness and Safety of Trokie^®^ Lozenges: A Standardized Formulation for the Buccal Delivery of Cannabis Extracts

**DOI:** 10.3389/fnins.2018.00564

**Published:** 2018-08-14

**Authors:** Kenton Crowley, Sieta T. de Vries, Guillermo Moreno-Sanz

**Affiliations:** ^1^Palliative Care Corporation, Huntington Beach, CA, United States; ^2^Department of Clinical Pharmacy and Pharmacology, University of Groningen, University Medical Center Groningen, Groningen, Netherlands; ^3^Abagune Research, Vitoria-Gasteiz, Spain; ^4^Phytoplant Research S.L., Córdoba, Spain

**Keywords:** cannabis, effectiveness, safety, adverse events, trokie^®^ lozenges, standardized, buccal administration, polyethylene glycol

## Abstract

Therapeutic use of cannabinoids, the main active ingredients of *Cannabis* *sativa* L., is often hindered by their limited bioavailability and undesirable psychoactivity. We conducted an observational study in December 2016 and another one in February 2018 to investigate respectively: (i) the effectiveness of Trokie^®^ lozenges, a standardized formulation containing cannabis extracts, to deliver cannabinoids via buccal absorption and (ii) its long-term safety. Participants were members of the Palliative Care Corporation health clinic, registered California cannabis patients, and had a diagnosis of chronic non-cancer pain. For the effectiveness study, 49 participants were asked to self-report pain perception before and after 1–12 weeks of taking Trokie^®^ lozenges, using an 11-point pain intensity numeric rating scale (PI-NRS). A mean reduction in PI-NRS score of 4.9 ± 2.0 points was observed. Onset of analgesia typically varied between 5 and 40 min, which seems consistent with, at least partial, buccal absorption. In the safety study, 35 participants were asked to complete a questionnaire about adverse events (AEs) associated with Trokie^®^ lozenges. AEs were reported by 16 subjects (46%), the most common being dizziness/unsteadiness (*N* = 7), bad taste (*N* = 5), and throat irritation/dry mouth (*N* = 4). None of the self-reported AEs resulted in a serious medical situation and most of them had limited impact on daily functions. Despite the AEs, 90% of participants reported being “satisfied” or “very satisfied” with the product. These observations suggest that buccal administration of standardized extracts via Trokie^®^ lozenges may represent an efficacious and safe approach to cannabis administration.

## Introduction

In recent years, many countries have passed legislation permitting the use of cannabis for medical reasons. This has given patients access to both herbal material and cannabis-based products, bypassing the strict regulatory procedures that usually apply to pharmaceutical development (Fitzcharles and Eisenberg, 2018). Further, legalization of cannabis for recreational purposes in several states of the Union as well as in internationally-relevant countries, such as Canada, conferrers cannabis an intriguing dual status, both as a pharmaceutical substance and a mere commodity, thus creating a complex regulatory scenario. Manufacturers of cannabis products, irrespective of their purpose, are met alike with the limitations that the lipophilic nature of cannabinoids, the main active substances in *Cannabis sativa* L., poses to their absorption and distribution in the human body (Huestis, 2005). Moreover, the psychoactive effects induced by Δ^9^-tetrahydrocannabinol (THC), the primary active ingredient present in cannabis, need to be: (i) minimized for patients to maximize the therapeutic index of cannabis medications, and (ii) carefully controlled for adult consumers in order to reduce both the risk of acute intoxication and the impact on public health and safety. Economic reasons also play a role since increasing the bioavailability of cannabinoids could allow for a reduction in the amount of active ingredient per dose, thus lowering the cost of the final product. Current efforts to achieve efficient, reliable dosing aim at exploring different routes of administration (e.g., pulmonary, oral, mucosal, transdermal) and delivery vectors (e.g., inhaling powders, nanovehicles) to optimize the pharmacokinetic profile of cannabinoids (van Drooge et al., 2005; Conte et al., 2017). Given the current lack of regulation in the medical and adult markets, cannabis-based products are readily available for the general population to acquire and consume without medical supervision. For some researchers, this represents a waste of valuable clinical information that could be extremely useful if adequately collected.

Sativex^®^ (USAN: nabiximols), an ethanol-based oromucosal preparation with 2.7 mg of THC and 2.5 mg cannabidiol (CBD) per spray, was the first cannabis product to attain regulatory approval as a pharmaceutical in 29 countries, having met the required standards of safety, efficacy and consistency (MacCallum and Russo, 2018). Nevertheless, it presents some adverse events (AEs) associated either with the pharmacology of cannabinoids (e.g., dizziness, drowsiness, dry mouth) or the detrimental effects of alcohol upon the oral mucosa (Scully, 2007). An alternative to ethanol could be muco-inert polymeric coatings such as polyethylene glycol (PEG), a hydrophilic, non-ionic, biocompatible polymer considered the gold standard in engineering mucus-penetrating surfaces. PEG grafting reduces adhesion to mucin fibers, allowing nanoparticles to quickly diffuse through the interstitial fluids enabling sustained mucosal drug delivery (Huckaby and Lai, 2018). Here, we have assessed the ability of a standardized cannabis formulation containing PEG (Trokie^®^ lozenges) to deliver cannabinoids via the buccal/oral mucosa as well as associated AEs.

## Materials and Methods

### Research Population

We conducted two observational studies with members of the Palliative Care Corporation (PCC), a health clinic in Huntington Beach, CA, which distributes Trokie^®^ lozenges. Admission criteria for the health clinic include: (i) willingness to incorporate cannabis into current medication regime; (ii) not having known allergies to cannabinoid drugs (dronabinol, nabilone, nabiximols); (iii) not having a cardiac arrhythmia; and (iv) not being pregnant. At the time of enrollment, members were asked to rate their pain on a pain intensity numeric rating scale (PI-NRS). This study was carried out in accordance with the principles of the Declaration of Helsinki and followed the relevant institutional and national guidelines. The study met the criteria set forth in 45 CFR §46.101(b) (4) and was therefore exempted from institutional review board (IRB) approval by Quorum Review IRB (Seattle, WA, United States).

### Cannabis Self-Administration Protocol

New members joining PCC, especially if cannabis-naive (which represent approximately 60%), were instructed on how to properly self-administer Trokie^®^ lozenges, embracing the “start low, go slow” motto proposed by clinicians for the use of herbal cannabis (MacCallum and Russo, 2018), starting with 1/4th of a 50 mg CBD lozenge (12.5 mg) two or three times a day for 3 days. After this initial period, members are coached by PCC clinical personnel on how to slowly adjust their intake of THC and CBD to achieve optimal symptom relief, with follow-up calls on days 4, 7, 14, and each time they place a new order, under the guiding principle of “primum non nocere” (Fitzcharles and Eisenberg, 2018).

### Preparation of Trokie^®^ Lozenges

The following 4-dose lozenges were available to members at PCC: 50 mg (only CBD), 40 mg (1:1 ratio CBD:THC), 64 mg (1:15 ratio CBD:THC), 120 mg (1:15 ratio CBD:THC), and 120 mg (only THC). Trokie^®^ lozenges were prepared as described in U.S. Patent No. 62/018,484 (Crowley, 2017). After preparation, batch samples were sent to a third-party laboratory for analysis (CannaSafe Analytics, Murrieta, CA Unites States). Batches were quarantined until laboratory results allowed release for packaging and labeling.

### Study 1: Effectiveness of the Delivery System

On December 2016, members of the PCC with a diagnosis of chronic non-cancer pain who had been enrolled for at least 12 weeks were invited to participate in a phone interview conducted by KC. The time frame (1–12 weeks) was selected to maximize the number of members actively enrolled in the clinic at the time of the study. Participants were asked: (i) to score their current pain on a PI-NRS from 0 to 10 (Farrar et al., 2001), (ii) answer a global self-assessment of feeling better, same or worse, and (iii) to estimate the onset of analgesia after taking Trokie^®^ lozenges. Participants using opiate medication were questioned about any modifications on their intake from the time of enrollment.

### Study 2: Assessment of Adverse Events

To assess the safety of Trokie^®^ lozenges, we conducted a survey on February 2018 among PCC members with chronic non-cancer pain that had been enrolled for a sustained period of time, between 4 and 60 weeks. This time frame was selected to capture AEs associated to mid- to long-term use of Trokie^®^ lozenges. Participants were asked to complete a questionnaire to report adverse drug reactions (de Vries et al., 2013), slightly modified to focus on AEs caused by Trokie^®^ lozenges.

### Analyses

Pain intensity was calculated as the difference between PI-NRS values reported on December 2016 and those reported at enrollment, and it is expressed as mean ± standard deviation of the mean. Significance of differences was determined using a paired *t*-test (PI-NRS before vs. PI-NRS after). Differences were considered significant if *P* < 0.05. Statistical analyses were conducted using GraphPad Prism Version 7.0 (San Diego, CA, United States). Patient-reported AEs were analyzed descriptively using IBM SPSS Statistics version 23 (Armonk, New York, NY, United States).

## Results

### Study 1: Effectiveness of the Delivery System

**FIGURE 1:**
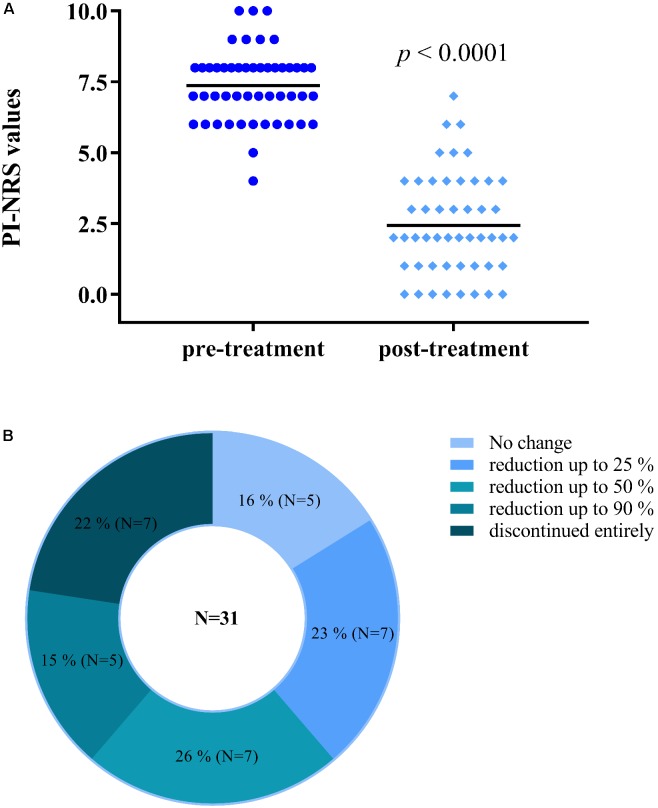
Participants using Trokie^®^ lozenges reported a mean reduction in pain intensity of 4.9 ± 2.0 points **(A)**, from a median value of 7.4 (pre-treatment, dark blue circles) to 2.4 (post-treatment, light blue diamonds) on an 11-point scale (0 = no pain, 10 = worst pain imaginable). Among 31 participants using opiates, 26 (84%) reduced in some degree or completely discontinued their use of opiate medication **(B)**.

A total of 49 participants (15 males/34 females) with an average age of 59.9 years completed the study (descriptive statistics in **Supplementary Table 1**). An average reduction in PI-NRS score of 4.9 ± 2.0 points (from 7.4 ± 1.3 to 2.4 ± 1.8) was observed (**Figure 1A**). Also, among 31 participants using opiates, 26 (84%) voluntarily reduced or discontinued their use of opiate medication (**Figure 1B**). These reductions were completed by participants with no reported symptoms of opiate withdrawal. Lastly, all participants reported feeling an improvement in their condition (100%) and an onset of analgesia between 5 and 40 min.

### Study 2: Assessment of Adverse Events

The adverse drug reaction questionnaire was completed by a total of 35 participants (7 males/28 females) with an average age of 65.3 years (descriptive statistics in **Supplementary Table 1**). Twenty AEs were reported by 16 participants (46%), with most reporting one single AE (*N* = 12; 75%) and four reporting two AEs. Descriptions given by participants corresponded to 7 AEs (with some of them relating to more than one AE) which were: dizziness/unsteadiness (*N* = 7), bad taste (5), throat irritation/dry mouth (*N* = 4), drowsiness/fatigue (*N* = 3), impaired consciousness/high feeling (*N* = 2), nausea (*N* = 1), and palpitations (*N* = 1). There were no reports of damage to the oral mucosa, gums, or teeth. None of the self-reported AEs resulted in a serious medical situation and most of them had only limited impact on daily functioning. Of notice, AEs associated with CBD-only Trokie^®^ lozenges were not related to any psychoactivity and were restricted to bad taste (4) and throat irritation/dry mouth (2). The remainder of AEs were associated with THC-containing lozenges, alone or in combination with opiates or other THC-containing products. Despite these AEs, 90% of participants reported being “satisfied” or “very satisfied” with the product (**Table 1**).

**Table 1:** Nature of the reported adverse events (AEs).

	N (%)
**First time experiencing the AE**	
Today	0 (0)
Yesterday	0 (0)
2–7 days ago	0 (0)
Between 1 week and 1 month ago	0 (0)
Between 1 and 6 months ago	1 (5)
Between 6 and 12 months ago	3 (15)
More than 12 months ago	16 (80)
**How much bothersome**	
Not at all	0 (0)
Only a bit	7 (35)
Somewhat	6 (30)
Quite a lot	6 (30)
Very much	1 (5)
**Influence daily functioning**	
None	10 (50)
Only a bit	4 (20)
Somewhat	2 (10)
Quite a lot	3 (15)
Very much	1 (5)
**Drug causing the AE**	
One drug, namely:	18 (90)
Trokie^®^ lozenges CBD	6
Trokie^®^ lozenges CBD and THC	5
Trokie^®^ lozenges THC	7
More than one drug	2 (10)
Trokie^®^ lozenges THC + Opiates	1
Trokie^®^ lozenges THC + THC capsules	1
**Participants’ certainty about the relationship between the reported AE and Trokie^®^**	
Very sure	18 (90)
Quite sure	2 (10)
Not very sure	0 (0)
Very unsure	0 (0)
**Satisfaction with Trokie^®^ lozenges**	
Very satisfied	9 (45)
Satisfied	9 (45)
Neither satisfied or dissatisfied	0 (0)
Dissatisfied	1 (5)
Very dissatisfied	1 (5)

## Discussion

Trokie^®^ lozenge is a cannabis-based product currently available in six states of the Union (Arizona, California, Florida, Iowa, Nevada, and Minnesota) and Puerto Rico. Two observational studies were conducted with California-certified cannabis patients to assess the effectiveness and safety of Trokie^®^ lozenges. First, we focused on the effectiveness of Trokie^®^lozenges to deliver cannabinoids, namely THC and CBD, through the buccal mucosa: *does it work in practice?* (Haynes, 1999). Our findings indicate that the use of Trokie^®^ lozenges is associated with a self-reported pain reduction in chronic, non-cancer pain patients, a condition for which the efficacy of cannabis has been previously described (Lynch and Ware, 2015). Of note, reported time to onset was between 5 and 40 min which, considering lozenges take 20–25 min to dissolve, seems consistent with, at least partial, buccal absorption (Karschner et al., 2011). However, it is reasonable to assume that it can also be swallowed with saliva. Correct placement in the mouth appeared to be critical for minimizing saliva production. Next, we aimed at assessing the safety of Trokie^®^ lozenges and the kind of AEs that could be associated with its long-term use: *is it safe to use?* Seven different AEs were reported by participants, one of them related to the organoleptic qualities of the product (bad taste) and the rest being common to other cannabis products containing THC, such as dizziness or dry mouth, none of which resulted in a serious medical situation and only had limited impact on daily functioning. Interestingly, our results are in strong agreement with a recent study performed in Israel on a large cohort of elderly (over 900 participants, 74.5 ± 7.5 years) reporting a reduction of pain levels from a median of 8 to a median of 4 on a scale of 0–10 after 6 months of cannabis treatment. Further, most common AEs were dizziness (10%), dry mouth (7%), and 18% of participants stopped using opiate analgesics or reduced their dose (Abuhasira et al., 2018). In our case, the proportion of participants reducing or discontinuing opiate analgesics was significantly larger (84%), similar to what has been previously found in a study based on patient self-reports (Reiman et al., 2017). This may be explained by the extensive use of opiate medication in the United States and the fact that many PCC members are seeking cannabis treatment because they “want to get off opiates and don’t want to get high” (personal communications with PCC staff).

Legalization of medical and recreational cannabis has bypassed the usual drug regulatory procedures in jurisdictions worldwide. Pending sound evidence for its effects in many conditions, physicians face the challenge of continuing to provide competent, compassionate care with an emphasis in harm reduction. Nevertheless, this regulatory scenario creates unprecedented opportunities to study the clinical impact of cannabinoids in human health and behavior. An illustrative example of how commodities can revolutionize the way we perform biomedical research are consumer physical activity monitors, which have raised enormous interest to physiology and psychopathology research because of their ability to measure activity continuously under real-life conditions and because they are already widely used by consumers (Wright et al., 2017). We believe the present results provide valuable information in terms of route validation, dosage selection and expected AEs, while cognizant of the severe limitations of our design due to the nature of the research, such as biased participant selection, lack of blinding or absence of placebo control. Therefore, these results should not be interpreted to establish any causality between the use of Trokie^®^ lozenges and the improvement in participants well-being. However, the findings support the need for conducting a phase 1 clinical trial to formally characterize the pharmacokinetic profile of Trokie^®^ lozenges in humans.

## Author Contributions

KC and GM-S designed the studies. KC coordinated the field research and conducted interviews. GM-S and SdV analyzed the data and generated results of the effectiveness and safety studies, respectively. GM-S wrote the manuscript with the aid of KC and SdV.

## Conflict of Interest Statement

KC is an inventor on U.S. Patent No. 62/018,484, describing buccal and sublingual cannabinoid formulations and method of making the same, which are the basis for the preparation of Trokie^®^ lozenges. The remaining authors declare that the research was conducted in the absence of any commercial or financial relationships that could be construed as a potential conflict of interest. The reviewer MDP and handling Editor declared their shared affiliation.

## Abbreviations

AEs: Adverse events
CBD: Cannabidiol
IRB: institutional review board
PCC: Palliative Care Corporation
PEG: polyethylene glycol
PI-NRS: Pain intensity numeric rating scale
THC: Δ^9^-tetrahydrocannabinol.
