# Supplementary material for: Self-Reported Effectiveness and Safety of Trokie® Lozenges: A Standardized Formulation for the Buccal Delivery of Cannabis Extracts
Source: Front Neurosci. 2018 Aug 14;12:564. doi: 10.3389/fnins.2018.00564 (PMC6102350; doi:10.3389/fnins.2018.00564)
Supplement: Supplementary file 1 [file Table_1.pdf]

Supplementary table. Descriptive statistics of the participants included in the two studies.

	Study 1. Assessment of the effectiveness of the delivery system	Study 2. Assessment of related adverse events (AEs)
Number of included participants	49	35
Number of females (%)	34 (69)	28 (80)
Average age in years (SD)	59.9 (10.9)	65.3 (13.7)
Number of opiate users (%)	31 (63)	12 (34)
Number of weeks taking Trokie® Lozenges	1-12	4-60
Most frequent diagnosed painful condition (N)*	Low back pain (15)<br>Osteoarthritis (15)<br>Migraine (7)<br>Fibromyalgia (6)	Joint pain (14)<br>Osteoarthritis (13)<br>Low back pain (9)<br>Migraine (5)

Of 49 participants in Study 1, 16 were also included in Study 2.

SD: standard deviation; * participants could present more than one diagnosed painful condition.
